# Optimising Stallion Semen Cryopreservation: Preliminary Insights Into Pre‐Centrifugation Extender Effects

**DOI:** 10.1111/rda.70135

**Published:** 2025-10-22

**Authors:** Bruna Merci de Zutter, Camila de Paula Freitas‐Dell’Aqua, José Antônio Dell’Aqua‐Junior, Gabriel Augusto Monteiro, Tiago Troncarelli, Frederico Ozanam Papa

**Affiliations:** ^1^ Department of Veterinary Surgery and Animal Reproduction School of Veterinary Medicine and Animal Science, São Paulo State University (UNESP) Botucatu São Paulo Brazil

**Keywords:** andrology, cryopreservation, freezing, sperm‐kinetics, stallion

## Abstract

This study evaluated the effects of cholesterol, pentoxifylline and casein, with or without skim milk, added to extenders during pre‐centrifugation on equine sperm cryosurvival. Seven ejaculates from four stallions (*n* = 28) were divided into four groups: SM (skim milk), SMP (SM + pentoxifylline), SMCho (SM + cholesterol) and ChoCa (cholesterol + casein). After centrifugation and freezing, sperm kinematics and plasma membrane integrity were assessed immediately and 30 min post‐thaw. SMCho and ChoCa showed superior results compared with SM and SMP. These findings indicate that cholesterol‐based extenders improve post‐thaw sperm quality when added before cryopreservation.

## 1 Introduction

Stallion spermatozoa are highly susceptible to oxidative stress, lipid peroxidation, cold shock and centrifugation, which impair motility, membranes and acrosomes (Aurich 2005; Peña and Gibb 2022; Sharafi et al. 2022). Extenders help buffer pH, provide energy and reduce bacterial and oxidative damage, but their formulation critically affects cryosurvival and fertility (Aurich et al. 2020; Peter et al. 2021; Spizziri et al. 2010). This study investigated the effects of cholesterol, pentoxifylline or casein‐containing extenders added before centrifugation on the viability of thawed stallion sperm.

## 2 Materials and Methods

### 2.1 Ethics Statement

This study was conducted in accordance with the principles of ethical animal experimentation, which was approved by the Board of Ethics in Animal Experimentation (Protocol No. 0508/2023) at UNESP School of Veterinary Medicine (Botucatu, São Paulo, Brazil).

### 2.2 Animals

Four healthy stallions (5–20 years) Quarter Horse, Brazilian Criollo Horse and Mangalarga Marchador Horse were selected during the breeding season (South hemisphere: September–October 2023).

### 2.3 Semen Collection and Evaluation

Twenty‐eight ejaculates were collected at 2‐day intervals using a Botucatu artificial vagina (Botupharma, Botucatu, SP, Brazil). Sperm concentration was determined with a Neubauer chamber. Each ejaculate was split into four aliquots and diluted (1:2) in one of four pre‐centrifugation extenders: SM (BotuSÊMEN; skim milk), SMP (BotuTURBO; skim milk + pentoxifylline), SMCho (BotuSÊMEN SPECIAL; skim milk + cholesterol) and ChoCa (BotuSÊMEN GOLD; casein + cholesterol). According to manufacturer information, the base composition is similar, with differences restricted to the addition of pentoxifylline, cholesterol or casein plus cholesterol.

Sperm kinematics were evaluated by CASA (HTM‐IVOS 12, Hamilton Thorne, Beverly, MA, USA) adjusted for stallions (Carneiro et al. 2018), recording total motility, progressive motility, percentage of rapid spermatozoa and average path velocity (VAP, μm/s). Plasma membrane integrity (PMI) was assessed by dual CFDA/PI staining under epifluorescence, classifying CFDA^+^/PI^−^ sperm as viable and PI^+^ sperm as damaged, following Harrison and Vickers (1990), with adaptations for equine semen (Brinsko et al. 2003; Larentis et al. 2018).

### 2.4 Cryopreservation of Semen

After evaluation, samples were centrifuged (600 × g, 10 min), the supernatant discarded and pellets resuspended in BotuCRIO (Botupharma, Botucatu, SP, Brazil) to a final concentration of 50 × 10^6^ sperm/mL. Aliquots were packaged in 0.5 mL straws, cooled at 5°C for 20 min, and frozen as described by Papa et al. (2008). Thawing was performed at 37°C for 30 s, followed by reassessment of sperm kinematics and plasma membrane integrity.

### 2.5 Statistical Analysis

Data were expressed as mean ± standard error and analysed with GraphPad Prism 6.0. Normality and homogeneity were verified by Kolmogorov–Smirnov and Levene's tests. Parametric data were analysed by repeated‐measures ANOVA followed by Tukey's test, and nonparametric data by Friedman's test followed by Dunn's test. Significance was set at *p* < 0.05.

## 3 Results

Cholesterol‐based extenders (SMCho and ChoCa) significantly improved sperm kinematics compared to the pentoxifylline‐based extender (SMP) across pre‐freezing, post‐thawing and 30 min post‐thawing evaluations (Table 1).

**TABLE 1:** Mean values ± standard error of the characteristics of the sperm kinematics in 28 ejaculates from four stallions at the pre‐freezing, post‐thawing and 30 min post‐thawing.

Moment	Characteristic	SM	SMP	SMCho	ChoCa
Pre‐freezing	Total motility (%)	90.1 ± 0.6^ab^	88.6 ± 0.9^b^	89.9 ± 0.7^b^	91.6 ± 0.5^a^
	Progressive motility (%)	39.5 ± 2.0^a^	39.7 ± 2.4^a^	41.1 ± 2.0^ab^	42.2 ± 2.0^b^
	Percentage of rapid sperm (%)	83.9 ± 1.0^a^	82.6 ± 1.2^a^	84.2 ± 1^ab^	86.2 ± 0.8^b^
	Average path velocity (mm/s)	143 ± 2.1^a^	134 ± 3.0^b^	138 ± 2.4^ab^	142 ± 2.4^a^
Post‐thawing	Total motility (%)	75.3 ± 1.8ab	73.9 ± 1.7a	78.1 ± 1.5b	78.9 ± 1.3b
	Progressive motility (%)	40.8 ± 2.6a	41.6 ± 2.6a	44.7 ± 2.3b	45.1 ± 2.3b
	Percentage of rapid sperm (%)	60.1 ± 3.2a	61.2 ± 2.8ab	64 ± 2.8ab	65 ± 2.5b
	Average path velocity (mm/s)	97.6 ± 2.2a	96.1 ± 1.8a	97.6 ± 1.9ab	101 ± 1.8b
30 min post‐thawing	Total motility (%)	66.5 ± 2.0ab	61.8 ± 2.6a	70.2 ± 2.0bc	71.9 ± 2.0c
	Progressive motility (%)	32.7 ± 2.6ab	31 ± 2.8a	36 ± 2.8b	36.6 ± 2.5b
	Percentage of rapid sperm (%)	52.2 ± 2.9ab	49.1 ± 3.5a	54.9 ± 3.1bc	57.8 ± 3.2c
	Average path velocity (mm/s)	92.5 ± 2.1a	87.3 ± 2.5b	90.9 ± 2.0ab	91.7 ± 2.2a

*Note:* ^ab^Different letters in the same line differ significantly from each other (*p* < 0.05).

Abbreviations: ChoCa, cholesterol and caseinate; SM, skim milk; SMCho, skim milk with cholesterol; SMP, skim milk with pentoxifylline.

Regarding sperm membrane integrity, the ChoCa group (76.4% ± 1.5%) demonstrated significantly better results (*p* < 0.05) at pre‐freezing compared to the SM (71.9% ± 1.5%), SMP (70.1% ± 2.0%) and SMCho (73% ± 2.0%) groups. At post‐freezing, the ChoCa (58.3% ± 1.4%) and SMCho (57.4% ± 1.9%) groups also showed superior membrane integrity (*p* < 0.05) when compared to the SM (49.2% ± 1.3%) and SMP (52.5% ± 1.9%) groups.

## 4 Discussion

The association between sperm kinetics and plasma membrane integrity (PMI) is predictive of fertility, with both parameters declining during cryopreservation, particularly during the critical cooling phase between 18°C and 9°C when lipid loss and metabolic instability compromise viability (Foster et al. 2011; Stephens et al. 2013; Spizziri et al. 2010; Papa et al. 2008).

The addition of pentoxifylline, although stimulating ATP production via cAMP and increasing initial motility, resulted in poorer post‐thaw performance. Similar findings in other species suggest that premature energy depletion and membrane destabilisation reduce velocity and linearity (Negri et al. 1996; Gil et al. 2010; Guasti et al. 2017; Gradil and Ball 2000).

In contrast, cholesterol‐containing extenders (SMCho and ChoCa) improved kinetic parameters and PMI, confirming the role of cholesterol in bilayer stability and protection against cold shock (Hartwig et al. 2014). The superior performance of ChoCa may be linked to sodium caseinate, which reduces cholesterol efflux mediated by binding proteins and contributes to motility preservation (Zahn et al. 2002; Campos et al. 2020). Physiologically, cholesterol incorporation occurs during epididymal maturation via binding proteins and lipoproteins that stabilise the membrane. In semen extenders, incorporation can be achieved through cholesterol‐loaded cyclodextrin (CLC), which promotes direct insertion, or through components such as sodium caseinate, which enhance retention and stability (Spizziri et al. 2010).

These findings align with Zanlorenzi‐Silva et al. (2025), who reported superior results for SMCho and ChoCa after passive refrigeration and conventional freezing, and with El‐Amrawi et al. (2021), who observed higher fertility in SMCho compared to SM, SMP and ChoCa. Importantly, the present study adds comparative evidence through a split‐sample design and evaluation at three time points (pre‐freezing, post‐thawing and 30 min post‐thaw), strengthening understanding of the effects of cholesterol and caseinate on equine sperm cryosurvival.

Although all ejaculates met CBRA (2013) standards, individual variability highlights the need for extender selection tailored to each stallion. Cholesterol‐based extenders, particularly when combined with sodium caseinate, demonstrated superior performance over skim milk‐based extenders, suggesting advantages for pre‐freezing centrifugation. Future in vivo fertility trials are warranted to confirm the mechanisms and validate their reproductive applicability.

## Author Contributions

Bruna Merci de Zutter: writing – original draft, investigation, visualisation. Camila de Paula Freitas‐Dell'Aqua: writing – review and editing, formal analysis. José Antônio Dell'Aqua‐Junior: conceptualization, methodology. Gabriel Augusto Monteiro: writing – review and editing. Tiago Troncarelli: translation. Frederico Ozanam Papa: conceptualization, methodology, supervision.

## Conflicts of Interest

The authors declare no conflicts of interest.

## Data Availability

The data that support the findings of this study are available from the corresponding author upon reasonable request.
